# Designing an ICT self-management service: suggestions from persons with type 2 diabetes

**DOI:** 10.1007/s12553-016-0176-9

**Published:** 2017-01-11

**Authors:** Cecilia Gardsten, Christina Mörtberg, Kerstin Blomqvist

**Affiliations:** 1 School of Health and Society, Kristianstad University, SE-291 88 Kristianstad, Sweden; 2 Department of Informatics, Linnaeus University, Vaxjo, Sweden

**Keywords:** Type 2 diabetes, Future workshop, ICT service, Chronic disease selfmanagement, Person-centred care, Participatory design

## Abstract

This paper reports the wishes and needs of people with type 2 diabetes (T2DM) for a future information and communication technology (ICT) self-management service to help manage their condition and their everyday life. Diabetes is a chronic disease affecting more and more people and placing increasing demands on health care. The self-management of diabetes includes instrumental and, decision-making skills and skills in managing daily activities, which may be supported by an ICT service. In this study we used a participatory design including two sessions of Future Workshop (FW) as part of a larger research project on the self-management of diabetes. Adults with type 2 diabetes participated in two FW sessions in which their expressed wishes and needs for an ICT service all fell under the broad category of Acceptance of the diagnosis, with three other suggestions; Trust in partnerships, Communication, and Individualized information. The participants’ experience of the FW as a democratic process and their appreciation of mutual learning contributed to these results, which are consistent with the aims of person-centred care.

## Introduction

Diabetes is a global health problem and one of a number of known chronic diseases with placing increasing demands on health care. Diabetes occurs in two different types: type 1 and type 2. Type 1 diabetes is characterized by a lack of insulin production and requires daily administration of insulin; type 2 diabetes mellitus (T2DM) is characterized by high blood glucose levels and, a relative lack of insulin. T2DM accounts for 90% of all diabetes in the world, and the number of people living with T2DM is increasing [1]. Although, a national study recently showed a modest decrease in the incidence of T2DM, the number of people living with T2DM in Sweden is still high. One explanation for this high prevalence is assumed to be the long life expectancy in Sweden [2, 3]. Considering the development of diabetes over time and increased demand of self-management, there is great potential to improve the lives for adults living with T2DM. Living with diabetes requires self-management and meeting challenge such as managing changes to daily habits, blood glucose testing, and compliance with medical prescription [4].

Self-management involves challenges related to diet, skin care, medication, and exercise [5]. Thus, skills in self-management are more complex than simply following recommendations based on standardized knowledge [6]. Self-management requires skills, decision-making, skills for managing daily activities [7] depending on blood glucose levels that have to be managed over both the short and the long terms [8]. Even when blood glucose measurements are within normal limits, prescriptions have to be followed to prevent any future complications [8]. Learning from other adults with T2DM may help patients deal with everyday life and improve self-management [9]. Although, healthcare practitioner attitudes, services and interactions are valuable for adults learning to manage diabetes, other adult patients’ expertise gained through their own experiences of living with diabetes are also important to help patients understand their condition and learn self-management [4, 6].

Self-management focussed on peer-support might enable adults with diabetes to share their experiences [10] through interaction in virtual environments; synchronous conversations have also been shown to promote learning of self-management [11]. Online-information and communication technology (ICT) services may enable individualized care as a complement to face-to-face dialogues [12, 13], and sharing experiences in dialogues with other with similar health problems in an online service have been found to be valuable for self-management [14, 15]. However, the value of online support groups seems to depend on the individual’s willingness to interact, share experiences, and deal with serious health conditions [16].

Research indicates a need for individualized healthcare education [10]. Self-management, problem-solving, and coping skills, and quality-of-life all deserve more attention in intervention research [17]. Solutions that support patients in managing their own disease management are important when limited health care resources are strained by increasing numbers of people living with diabetes [18]. Even though research indicates that ICT services can facilitate self-management, several services are built to suit the needs of medical and health-care workers rather than individual patients needs and wishes [10]. An online technology service may be an complement to allow individuals to fulfil varying and specific needs [19, 20]. Future technologies are faced with the challenge of providing holistic, user-friendly, integrated, and individualized services [21, 22]. User-centred or participative design approaches is a way to involve users and to incorporate their requirements in systems design [23, 24]. To involve users to have a say in design of an ICT service are closely related to the foundations and principles in person-centred care [25]. The person-centred care model with an individual perspective has had a growing impact on health care [26] and constitute a tool for self-management. The principle of person-centred care aims to see the person as an individual and consider the whole person, taking into account each individual’s qualities, abilities and interests. Person-centred care as a systematic and consistent approach may satisfy the individuals’ unique needs in caring and self-management. Person-centred care begins with personal narrative for establishing a trustful and equal partnership based on shared communication [27].

The aim of this study was to identify adult patients’ wishes and needs for an ICT self-management service to facilitate their everyday life and to deal with T2DM. Therefore we report on both the participative research process and the participants’ suggestions for the design of an ICT self-management service. This paper is a second step of a larger project aimed to design an ICT self-management service built on person-centred framework for adults with T2DM by involving people with T2DM (see Gardsten, Blomqvist et al. 2015 unpublished data).

## Participatory design

Participatory Design (PD) was used in this study to involve adults with T2DM, diabetes nurses and researchers in the design process for an ICT service. The involvement of various stakeholders enables cooperation, facilitates patient empowerment, and takes into consideration how adults with T2DM manage their everyday activities [28, 29]. PD was established in 1970 with the aim to democratize both the working life and the design process [30]. PD has evolved since the 1970s, but its basic principle continues to be to give those who will be affected by a tool or programme such as an ICT system an equal say in its design [30, 31]. The various stakeholders in a PD project cooperate through participative methods and techniques, and their shared experiences and cooperation are necessary to the generation of the vision of the proposed system and the requirements necessary to its design [28]. Mutual learning, is also a core element in PD. Participatory methods such as FW, storyboards, scenarios, and cartographies enable cooperation and may facilitate mutual learning [28, 32]. Other guiding principles in PD are equalized power relations, democratic practices, and situation-based actions. Situation-based action in this research meant that we needed to pay attention to people’s knowledge and expertise about their own activities to deal with T2DM [31]. In an organization as complex and hierarchical as the health care sector, which involves various practitioners with well-defined boundaries, it is necessary to adopt a pragmatic approach in conduction a PD project with chronically ill people [29]. In the context of this paper, PD and person-centred care are considered as closely related approaches with a shared foundation where individuals interact about personal issues. We chose to use a PD in this study because our aim was to identify the wishes and needs of adults’ with T2DM for a future ICT self-management service. This was also in keeping with the main research project’s participative approach in creating discussions between various stakeholders to exploring their experiences of T2DM.

### Future workshop: a participative method

Future Workshop (FW) is one of several methods used in PD to involve participants in democratic practices to contribute to changes in their everyday lives [28]. The method was introduced in the 1980s to engage citizens by giving them a say in community decisions [31, 33], allowing various participants with alternate understandings to be heard and to engage with each other [31]. The facilitator acts as moderator, to allow all participants the opportunity to be heard and to encourage them to provide new ideas [28]. The purpose of an FW is first to create a common understanding, and based on that understanding to suggest a vision, in this case – wishes and needs for a future ICT service built on person-centred care for people with T2DM. The method consists of five phases: preparation, critique, fantasy, realization, and follow-up. The *preparation phase* includes planning the theme, implementing the ideas and decisions of a time table, and deciding who should be invited. This phase also includes decisions about an appropriate place for the FW and amenities such as refreshments. The aim of the *critique phase* is to identify problems and create a joint understanding of the current situation to be used in the following phases and activities. At the end of the critique phase participants discuss and evaluate different suggestions to create common ground and then vote upon their importance. In the *fantasy phase* the participants visualize how they wish their challenging situation could be improved. These ideas are generated without any restrictions. The fantasy phase ends similarly to the critique phase, with different ideas evaluated in discussion and ranked by votes. These proposals will then form the basis of a vision to be used in the next phase. In the *realization phase* the participants aim to make the vision concrete through suggestions of how to attain it. In the *follow-up phase* a written report summarising the process and the participants’ recommendations is circulated to the participants with an invitation for their involvement in further work towards their empowerment as recommended by Bødker et al. [28].

### Facilitating empowerment

A central principle of PD is encouraging participants’ empowerment through their participation in design processes [28]. Empowerment is often described in terms of relationships between individuals and can be achieved by mutual sharing [34] in a learning process [35]. Empowerment is based on people having a voice and the right to participate in influencing their environment. Being heard not only motivates people to act, but also gives them responsibility for changing their everyday lives to improve their health [19, 20, 35, 36]. People who collaborate in decisions that affect them are better equipped to participate in decision-making about their own self-management, and healthcare practitioner may therefore spend less time in efforts to motivate them [36]. By engaging people and helping them to become autonomous and to make independent decisions, healthcare practitioners may then have more time to listen, to take people daily lives into account, and to provide more individualized care [37]. However, structural barriers to empowerment are challenges that people in need of healthcare have to adapt to. Such examples are practitioners priorities and rules in a healthcare organization divided into different units [38]. Thus, access to different services is probably a precondition of self-decision making to facilitate individuals in the process to achieve empowerment [39]. Further research are required to make use of potentials of ICT to enable to support and to sustain peoples empowerment in care and healthcare [40].

## Research design

The main research project started in 2011 with adults with T2DM, diabetes nurses and researchers in an area in south of Sweden. Repeated sessions with focus groups were used in the first part (Gardsten, Blomqvist et al. 2015 unpublished data) and in this part the participative method was used to invite adults with diabetes and diabetes nurses to suggest wishes and needs for a prospective ICT service. The rationale for using a participative method builds on the main project’s foundation in person-centred care principles to involve various participants in discussions for exploring their experiences and ideas.

### Participants

The eleven adults with T2DM in the first part of the research were invited orally and in writing by a diabetes nurse to participate in the FW. A purposive sample was made at the start of the project in 2011 to reach adults with T2DM from a district health care centre and from a diabetes hospital clinic. Both recently diagnosed adults (≤ 3 years) and adults with experience of diabetes (≥ 5 years) were invited because they may have different desires for self-management. The adults with T2DM who participated in focus groups in the above-mentioned study were invited to participate in this part and the final sample was two adults recruited from the district health care centre and three adults from the diabetes hospital clinic. The age of the three women and two men ranged from 42 to 74 years, their experiences of diabetes from 3 to 10 years.

The FWs involved also two diabetes nurses, two researchers in nursing science (first and last author), one researcher in informatics and one researcher in computer science. The diabetes nurses did not participate in generating suggestions; they were involved to contribute their health care perspective on T2DM. The FWs were facilitated by a main facilitator (first author) and an assistant facilitator (last author). The role of the main facilitator was to introduce, lead, and summarize the sessions; the assistant facilitator supported the main facilitator, contributed to dialogues, made observations, and took notes for asking questions and giving feedback. The facilitators’ responsibilities were also to structure the overall process and to take an active role in helping participants to suggest creative ideas without being dominant in anyway. The table shows the participants in the recruiting process and their participation in each FW phase (Table 1).

**Table 1:** Participants in the recruiting process and participation in each FW phase

Recruiting process	Participants
Invited adults from previous study	11 adults with T2DM
Adults who confirmed their participation	6 adults with T2DM
Future Workshop phases	
The preparatory phase	2 diabetes nurses
	4 researchers
The fantasy phase	5 adults with T2DM
	2 diabetes nurses
	2 researchers
The realization phase	4 adults with T2DM
	2 diabetes nurses
	2 researchers

### Ethical considerations

The aims of the study were initially discussed with the diabetes nurses. The invited adults with T2DM were then informed orally and in writing about the aims, and that their participation would be voluntary and they could withdraw from the study whenever they wished without no explanation. Written informed consent was collected before each FW. Confidentiality and anonymization of the participants’ quotes were assured. The participants were informed in accordance with Bødker et al. [28] that an action plan would be formulated and that they would be asked to take part in the follow-up phase to influence the ICT service. The study was conducted according to the Helsinki declaration [41] and ethical permission was obtained from the Regional Ethical Review Board in Lund, Sweden (Dnr 2012/6). A request for permission [42] was also obtained from the two health care clinics where the participants were recruited.

### Data analysis

**Fig. 1:**
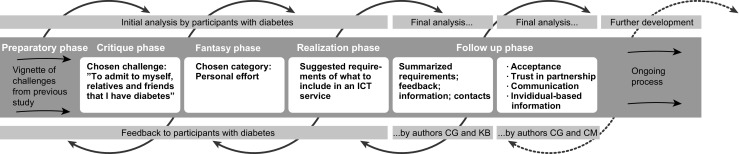
The outcomes of the FW phases including the analysis process

The initial analysis was performed in each FW session by the adults with diabetes themselves when vignettes, keywords, and topics were discussed, evaluated and assessed. Figure 1 shows how the empirical material was analysed as an ongoing process in several iterations, initially by the adults with diabetes, and later by the three authors. In addition, the outcomes of each FW phase were reported back to the participants with diabetes as an ongoing process to influence following research and outcomes.

The first and the last author analysed the outcomes from the realization phase by categorizing and summarizing the written material in the follow-up phase. The material was analysed in relation to the aim and resulted in concrete proposals related to nine categories of demands for a self-management services. The final analysis was conducted by the first and second author (CG and CM) through a qualitative content analysis in accordance with Lichtman [43] to search for patterns and examples of wishes and needs. The initial coding was done by the initial and summarizing analyses in FW procedure. The first and second author then read and discussed the written data repeatedly to get a sense of the whole. The data were read and rewritten to categorize excerpts with similar meanings. Finally, the categories were condensed into a main theme with three sub-themes. The post-it notes, whiteboard notes, field notes, summary analysis, and audio recordings were used to remember details of the FW and choose quotes to exemplify the participants’ ideas.

## Future workshop

The two FW sessions were held in October 2013 in a conference room at the hospital in the area where the adults with diabetes were recruited. Two FW sessions were planned, the first session included a sample of five adults with T2DM, two diabetes nurses, a main facilitator (first author) and an assistant facilitator (last author). The session started with the introduction of the participants, and, information about the aim and process of the FW; questions and discussions followed. A welcoming climate was created arranging the room to allow all participants to see each other and by providing refreshments. The two sessions were audio recorded and lasted about two hours each. The participants sat around a table, and those with T2DM were able to move to the whiteboard to paste post-it notes with keywords to vote on various options.

### Preparation phase

The preparation phase of the FW began with the involvement of the adults with T2DM in the focus group discussions, Gardsten, Blomqvist et al. 2015 unpublished data. Ethical permission was also obtained in the preparatory phase, and the FW method was demonstrated by the second author (informatics researcher) to the diabetes nurses and other researchers.

### Critique phase

The first part of the critique phase was replaced in this study by a presentation of the five vignettes created through a tentative analysis of the focus group discussions in the first part of the main study. The following five vignettes from previous study were presented:

1. “Support for shopping in grocery stores”
2. “Facilitate care contacts – to maintain good relationships”
3. “Understand relationships – and be able to deal with blood sugar levels and exercise and understand results”
4. “Understand the connection between and cope with stress and blood sugar”
5. “Understand the connection between and cope with meals and blood sugar”

Each vignette from the previous study was a short story about challenges in the self-management of diabetes such as understanding the relationship between blood glucose levels and stress. The critique phase continued with discussions of the vignettes to find topics to be used in the workshop. The participants with diabetes were not satisfied with the vignettes presented, and suggested an additional topic to those created in advance. This sixth topic was *Admitting to myself, my relatives, and my friends, that I have diabetes*. The critique phase continued with a discussion aimed at deciding the most important challenge to be used in the fantasy phase. The adults with diabetes decided through a series of votes on the sixth vignette *To admit to myself, relatives and friends that I have diabetes*. This topic was then primary in the subsequent fantasy phase. The discussions and shared understanding about the sixth vignettes constituted one part of the initial analysis by the participants with diabetes.

### Fantasy phase

**Fig. 2:**
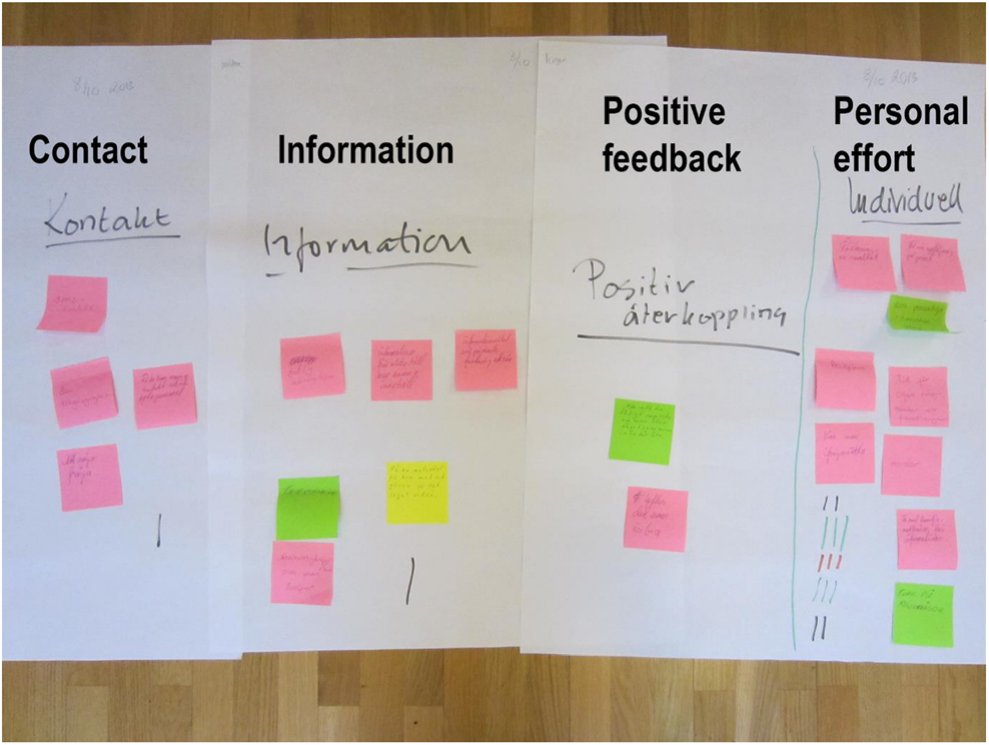
The four categories generated in the fantasy phase (translated by the authors). The category *Personal effort* contains the following keywords: “Explanation of results”, “Follow-up in writing”, “Importance of individualized, rather than general, information”, “Acceptance”, “Time for questions, ideas, and thoughts”, “Possibility to ask questions”, “Mentor”, “Inclusion of family formation sessions”, “Get replies to supplementary questions”

In the fantasy phase the adults with diabetes brainstormed about how an ICT service could help them admit to themselves, their relatives, and their friends that they have diabetes. Because these participants were hesitant at the start, the assistant facilitator used an example as an icebreaker to help them generate ideas. Then, each participant with diabetes wrote keywords on post-it notes and, pasted them on the whiteboard to share with other participants with diabetes to suggest ideas for a services to facilitate self-management. The ideas were discussed among all participants, evaluated by the participants with diabetes, and clustered into four main categories: *Contacts*, *Information*, *Positive feedback* and *Personal effort*. This phase ended with a vote and the decision to move the category of *Personal effort* to the realization phase (Fig. 2). Finally the facilitator summarized the fantasy phase. The first FW session ended with this. The brainstorming discussions and the shared understandings of the keywords constituted the initial analysis of the fantasy phase.

### Realization phase

The realization phase was held two weeks after the fantasy phase in October 2013. One adult with diabetes was unable to participate in this session. Four adults with T2DM, two diabetes nurses, two facilitators, and one computer science researcher involved in developing the ICT based self-management service participated. The session began with a summary of the critique and fantasy phases. An open climate was created and the participants with diabetes shared their own experiences of the category *Personal effort*. The FW is a method to collect participants shared decisions and choices for further work. Thus, an additional part of the initial analysis, was performed in the realization phase when the keywords from the selected theme *Personal effort* were discussed, evaluated, and assessed to elicit new topics. The keywords were critiqued, and adapted to the participants’ real life conditions. The adults with diabetes decided to exclude suggestions that they thought would not be feasible in the future. The realization session ended with suggestions for required aspects of an ICT self-management service such as individual written information and feedback, contacts with a person with experience of diabetes, and the involvement of family. These suggestions will be incorporated in the main project in an action plan to design an ICT self-management service. After the realization phase the first and the last author (CG and KB) categorized and summarized the suggestions as a part of the follow-up phase. The summary revealed that feedback, information and contacts were essential.

## Results – design suggestions

The final design suggestions were based on the category *Personal effort* and the outcome of the realization phase. The themes extracted from the final condensation of the created categories in previous iterations were the participants’ suggested wishes and needs for a self-management ICT service. This resulted in the main suggestion of *Acceptance,* with the three supporting suggestions *Trust in partnership; Communication; and Individualized information*. *Acceptance* was felt to be a necessary prerequisite to striving for self-management. Thus, these design suggestions are closely related to a person-centred framework with its focus on the patient and the patient’s perspective.

### Acceptance

The main suggestion, acceptance of the diagnosis T2DM, made it possible for participants to make their own decisions in everyday life. The diagnosis of diabetes came as a surprise to some participants, and these participants required a longer time to reach acceptance and adapt to their new life situation. The three supporting suggestions were all related to acceptance of the disease. Acceptance of the disease contributed in turn to skills, such as the ability to be involved in a trusted partnership that made it easier to communicate. Acceptance also made it easier to follow recommendations, understand and interpret individualized information for making decisions, and become capable of developing skills in self-management.

#### Trust in partnerships

This suggestion included various kinds of trust. Trust was a prerequisite for assimilating information and being willing to communicate. The initial step of building trust during health care meetings was seen by participants as a basic condition for partnership. Confidence in health care practitioners was required when asking for advices about diabetes and self-management. Participants also said that trust was important to cooperating in problem solving and to following advices with confidence. Trust in supportive relationships with healthcare practitioners and with relatives or friends was seen as important in the management of everyday challenges.

*I have to be able to conduct a dialogue based on the information.*

#### Communication

The second supportive suggestion of an ICT self-management service was the provision of options for communication, which were rated as important to facilitating self-management. The participants with diabetes suggested including opportunities to discuss everyday issues with healthcare practitioners and to clarify advice they had received from healthcare about medication and dosages that they may not have been able to fully understand in an awkward or rushed consultation. Participants with diabetes also suggested having an adult who had lived with T2DM for some years as a mentor who could questions and offer encouragement and insights on how to live with the disease. Hearing the life experiences of other adults with diabetes was perceived as valuable, because the disease causes different reactions and patterns in different people. Although it may offer different solutions, advice from other adults with diabetes was perceived as positive and comforting. Participants with diabetes recognized that they would have to make their own decisions about self-management and how to live.

*The big part is to talk to someone with experience. You don’t know at all what it means when you get the information … a mentor can tell you how to eat, how you live out there in society, all that.*

Another suggestion was that family members would be able to be involved on their own terms to get information and communicate. It may contribute to a supportive relationship with relatives if family members understand the condition and can ask questions of the health care practitioner. These options for communication might help the adult with diabetes to accept their diabetes more easily, make healthy decisions, and change to new habits.

*When you’ve received the diagnosis, then you have a meeting so that everyone’s clear about the situation* [short break] *everyone in the family gets to ask questions.*

#### Individualized information

Individualized information was the final supportive suggestion of an ICT self-management service. The individual written suggestions for information were refined through several concrete design suggestions. Individualization was considered a basic condition for making patient information about diabetes, such as the causes and effects of their own blood glucose levels, easier to understand. The participants with diabetes identified individualized written and verbal explanations as necessary to understanding their past and how they can manage their diabetes in the future, for example how their blood glucose level fluctuated over a specific time.

*It’s like, not just what’s happened, but also information about what you should change in order to get a better result.*

To facilitate understanding individual blood glucose levels, two concrete visualizations were suggested for inclusion in an ICT service. One was the use of a pie chart in colours to illustrate high (red), normal (green) and low (purple) blood glucose levels. The second suggestion was for a graph in which dots above a horizontal line illustrate a high blood glucose value and dots below the line illustrate a low value. One participant offered her experience of medication as an example of individual requirements. Her blood glucose test showed that she was well, but she felt sick. She left the healthcare visit and continued her medication without consulting her doctor.

*The last time was at the doctor’s, everything was just perfect and good* [on the tests]. *I felt like shit, but I just had to leave and go on.*

During this period she happened to forget her medication while travelling, but she felt well and was relieved to have forgotten the medicine. These examples show that the patients’ experience of their own health are not always consistent with measured blood glucose values. The participants’ stories highlighted individual experiences of diabetes, including various needs for written and verbal information.

## Discussion

The aim was to identify the wishes and needs of adults with T2DM for a future ICT self-management service to facilitate their everyday life and to deal with the disease. Our findings showed that the priority suggestions generated in the FW were the main suggestion *Acceptance* and its three supporting suggestions, *Trust in partnership, Communication, and Individualized information.* Acceptance of the diagnosis was a prerequisite for managing diabetes successfully. Acceptance of the diagnosis also made the participants accept information, learn about their condition, and understand how to personally manage in their everyday lives. *Trust in partnerships* with caregivers and C*ommunication* facilitated that acceptance and understanding of the disease.

An ICT service may also include a feature to facilitate the development of trustful partnerships. The participants with T2DM needed trustful partnerships as a tool to succeed in their self-management. The outcome of this research showed that trustful partnership between adults with diabetes and their healthcare practitioners was a condition for beneficial health dialogues. Trust was highlighted by participants as a requirement for asking questions about such things as medication and dosage. They argued that trustful and supportive relations facilitated their acceptance of diabetes and their ability to learn self-management. Trustful partnership between individuals and healthcare practitioners are also included in previous research as a condition for person-centred care [27]. An ICT service may include different communication channels as a complement to face-to-face meeting, to increase the opportunity for asking questions and getting answers about current self-management situations.

Adults with T2DM need access to communication services to facilitate their development in self-management. The participants with diabetes in this study reflected that both their different and similar experiences of diabetes were valuable and suggested that an exchange of experiences would enhance their empowerment in everyday life. The adults with diabetes wanted to be able to ask the advice of a fellow patient in a similar situation when a question arose. They considered mentorship by a fellow patient in addition to the healthcare practitioner’s services. Previous research has confirmed that interaction with a peer positively influences the learning process [11]. A peer-support service may meet self-management demands and enable adults with diabetes to share their experiences with others [18]. The value of communication in mentorship is supported by Mol [37] who claimed that healthcare practitioners often lack the time to listen to individuals and take their everyday lives into account. Exchange of experiences should be more highly valued as an important tool for gaining insights into the self-management of diabetes.

However, the suggested online peer-support service includes also ethical dilemmas. On the one hand, the PD approach used in this research implies that participants with TDM2 are treated as experts and PD’s ethical stand includes awareness and accountability for design suggestions [24]. On the other hand, a peer peer-support service governed by patients may include advices that are not in accordance with medical recommendations. Disadvantages of peer-support are also reported in previous work, such as patients fear, caused by reading about negative experiences of a disease [44–46]. In addition, patients had concerns about the quality of the information available in shared peer-support services [44, 45]. To deal with possible incorrect advices can e.g. be to automatically flag keywords that pose a risk for inaccurate advices or to ask peers to flag advices they discover are not reliable [45]. Further, the peer-support may also include a moderator [44] namely a competent person with solid experiences of TDM2 who in addition to general responsibilities may control whether peer-advices follow medical recommendations [43, 45].

A future ICT self-management service may include different options for communication with the intention of supporting and facilitating self-management. Adults with T2DM need different options of individualized information, for example to illustrate and explain the individual fluctuated blood glucose level. The participants with diabetes involved in this research suggested individualized information in writing and illustrations as a complement to verbal and general information. Previous research has indicated that an ICT service can offer advantageous access to different services for individual demands [39] and online programs are a useful resource for adults with diabetes [13]. Individual needs are dynamic and effort is required to provide appropriate clinical treatment, information, and support for diverse issues [10]. Hence, a service to facilitate individual demands for self-management also makes great demands on the health care system.

The participants with diabetes in this study acted as experts and contributed suggestions that fall within the patient education and shared partnership requirements of person-centred care. Person-centred care includes the individual’s narrative, trustful partnership, and shared and documented dialogues in healthcare. The intention of person-centred care is to improve patient empowerment by satisfying individual needs, preferences, and values in caring and self-management [27]. The results of this study were confirmed by previous research showing that empowerment was achieved by asking questions in a trustful mutual relationship [19, 20, 34, 36]. Ownership of the self-management process is important in decision making and contributes to patients’ independence, skills, and responsibility for actions that affect their lives [19, 20]. Access to adequate healthcare services has also been shown to be crucial for self-management and support of empowerment [39]. The priority suggestions for a self-management service suggested in this study may be delivered to meet different needs through an ICT service that encourages a person-centred approach.

As a participative method FW enabled the participants with diabetes to contribute to the awareness of the importance of collaboration. The participants’ various perspectives on self-management were communicated and negotiated in a reflective learning process in the participative workshop. Reflections and insights from the previous focus group study may also have influenced the participants with diabetes and contributed to their awareness, resulting in the sixth theme in addition to the themes presented by the researcher. Participants with diabetes expressed an awareness that exchanging experiences with others and affirming each other’s wishes and needs contributed to mutual learning. The participants’ enthusiasm and ability to participate in the research, generated conditions for mutual learning and they seemed to appreciate the cooperation and the sharing environment provided by the participative method, which allowed them to generate ideas and support each other [28]. Peoples’ experiences and knowledge about praxis is valuable in a research or development project when they will be affected of the improvement [20]. Involvement with others enables and creates awareness of different issues and may influence patients’ daily life. Thus, it is important to engage people in current issues. The FW method contributed to this research both through the findings proposed content for an ICT self-management service and by bringing attention to the advantages of mutual learning between participants. An incidental finding was that the FW method may also be useful for further collaboration to improve existing systems.

The priority suggestions for an ICT self-management service generated in this study were consistent with, and thus might encourage, a person-centred approach to care. However, the suggestions need to be explored in further research. Ethical questions must also be considered before it will be possible to provide an ICT service. The suggestions of individualized information will mean that a large amount of information will have to be offered in the ICT service. Hence, the design and maintenance of an ICT service will requires substantial resources from the healthcare sector, but this should be balanced against the benefits of self-management for adults with T2DM and possible benefits to the health care sector such as released time for other work.

### Conclusions

The findings showed that the ICT self-management service need to offer: different communication channels, possibilities for exchanging experiences and written and visualized individualized information. The suggested wishes and needs for an ICT self-management service turned out to be consistent with the description of person-centred care in terms of self-management in diabetes. The FW-method contributed to participants’ experiences of being the expert of oneself. Such experience might improve empowerment and contribute to pay attention of advantages in mutual learning.

### Strengths and limitations

The preparatory phase of this study was begun in an earlier stage of the research project. Thus, the FW in current study is a part of an extended process. The extended FW process with several collaborations seemed to contribute to mutuality and trust. A telling example of trust was that the participants felt comfortable questioning the proposed themes and adding the sixth theme in the critique phase. Recurring collaborations with involved stakeholders in an FW contribute to research development and work to drive the processes of change. Other strengths of the FW are its flexibility and ability to be modified. Weaknesses of the method are that it can be challenging to create ideas in some parts and the method might be experienced as time-consuming. Nevertheless, it may be worthwhile to spend more time with stakeholders when planning changes if resource savings might be identified before an implementation.

Because the research took place in a restricted geographical area, a further limitation was that participants from different ethnic groups were missing. The small sample of only five adults with T2DM from the previous study may be because other patients were reluctant or unable to spend two evening hours for each the two FW sessions. The pleasant atmosphere in the FW sessions may in part have been due to most participants having met each other earlier and formed bonds of mutual reliance. Although some of the participants had not met each other previously, they quickly formed a small and confident group and held comprehensive discussions. Collectively the group created a pleasant atmosphere, shared their experiences, and appreciated interacting with each other. The participation of the research in computer science as an observer in the second session did not seem to affect the discussions of the group. The diabetes nurses and the researcher in computer science also respected the adults with diabetes as the main participants. The facilitators’ main responsibility was to ensure that an equal process was achieved and maintained in the FW sessions. The facilitators gave the participants with diabetes the opportunity and power to make decisions such as adding the sixth theme.

## References

[1] World Health Organization. Diabetes: Fact sheet. Geneva. WHO. 2015. http://www.who.int/mediacentre/factsheets/fs312/en/ Accessed 2014–06-04.

[2] Jansson S, Fall K, Brus O, Magnuson A, Wändell P, Östgren C (2015). Prevalence and incidence of diabetes mellitus: a nationwide population-based pharmaco-epidemiological study in Sweden. Diabet Med.

[3] Norberg M, Danielsson M (2012). Overweight, cardiovascular diseases and diabetes health in Sweden: the National Public Health Report 2012. Chapter 7. Scand J Public Health.

[4] Vincent D, Clark L, Zimmer LM, Sanchez J (2006). Using focus groups to develop a culturally competent diabetes self-management program for Mexican Americans. The Diabetes Educator.

[5] Mars GM, Proot IM, Janssen PP, Van Eijk JTM, Kempen GI (2007). How do people with COPD or diabetes type 2 experience autonomy? An exploratory study. Journal of Disability and Rehabilitation.

[6] Thorne SE, Ternulf Nyhlin K, Paterson BL (2000). Attitudes toward patient expertise in chronic illness. Int J Nurs Stud.

[7] Moser A, van der Bruggen H, Widdershoven G (2006). Competency in shaping one’s life: autonomy of people with type 2 diabetes mellitus in a nurse-led, shared-care setting; a qualitative study. Int J Nurs Stud.

[8] Peel E, Douglas M, Lawton J (2007). Self monitoring of blood glucose in type 2 diabetes: longitudinal qualitative study of patients’ perspectives. BMJ.

[9] Deakin T, McShane CE, Cade JE, Williams R. Group based training for self-management strategies in people with type 2 diabetes mellitus. Cochrane Database Syst Rev. 2 2005. 10.1002/14651858.CD003417.pub2 15846663

[10] Ma C, Warren J, Phillips P, Stanek J (2006). Empowering patients with essential information and communication support in the context of diabetes. Int J Med Inform.

[11] Okita SY, Bailenson J, Schwartz DL, editors. The mere belief of social interaction improves learning. Proceedings of the Twenty-ninth Meeting of the Cognitive Science Society; 2007.

[12] Eysenbach G (2001). What is e-health?. J Med Internet Res..

[13] Ramadas A, Quek K, Chan C, Oldenburg B (2011). Web-based interventions for the management of type 2 diabetes mellitus: a systematic review of recent evidence. Int J Med Inform.

[14] Dumaij ACM, Tijssen ECG (2011). On-line health companion contact among chronically ill in the Netherlands. Heal Technol.

[15] Wicks P, Massagli M, Frost J, Brownstein C, Okun S, Vaughan T et al. Sharing health data for better outcomes on PatientsLikeMe. J Med Internet Res. 2010;12(2). doi:10.2196/jmir.1549. 10.2196/jmir.1549 PMC2956230 20542858

[16] van Uden-Kraan C, Drossaert C, Taal E, Lebrun C, Drossaers-Bakker K, Smit W (2008). Coping with somatic illnesses in online support groups: do the feared disadvantages actually occur?. Comput Hum Behav.

[17] Norris SL, Engelgau MM, Narayan KMV (2001). Effectiveness of self-management training in type 2 diabetes. Diabetes Care.

[18] Arsand E, Tatara N, Østengen G, Hartvigsen G (2010). Mobile phone-based self-management tools for type 2 diabetes: the few touch application. J Diabetes Sci Technol.

[19] Bergvall-Kåreborn B, Howcroft D, Ståhlbröst A, Wikman AM. Participation in living lab: Designing systems with users. Human Benefit through the Diffusion of Information Systems Design Science Research. Springer; 2010. p. 317–26.

[20] Jansson M, Mörtberg C, Mirijamdotter A, editors. Participation in e-home healthcare@ North Calotte. Proceedings of the 5th Nordic conference on Human-computer interaction: building bridges; 2008: ACM.

[21] Koch S (2010). Healthy ageing supported by technology-a cross-disciplinary research challenge. Inform Health Soc Care.

[22] Plsek PE, Greenhalgh T (2001). The challenge of complexity in health care. BMJ.

[23] El-Gayar O, Timsina P, Nawar N, Eid W (2013). A systematic review of IT for diabetes self-management: are we there yet?. Int J Med Inform.

[24] Robertson T, Simonsen J, Simonsen J, Robertson T (2012). Participatory design: an introduction. Routledge international handbook of participatory design.

[25] Wildevuur SE, Simonse LW (2015). Information and communication technology-enabled person-centered care for the “big five” chronic conditions: scoping review. J Med Internet Res..

[26] McCormack B, Karlsson B, Dewing J, Lerdal A (2010). Exploring person-centredness: a qualitative meta-synthesis of four studies. Scand J Caring Sci.

[27] Ekman I, Swedberg K, Taft C, Lindseth A, Norberg A, Brink E (2011). Person-centered care—ready for prime time. Eur J Cardiovasc Nurs.

[28] Bødker K, Kensing F, Simonsen J (2004). Participatory IT design : designing for business and workplace realities.

[29] Kensing F, Strand DL, Bansler J, Havn E, editors. Empowering Patients: PD in the Healthcare Field. PDC; 2004.

[30] Bjerknes G, Bratteteig T (1995). User participation and democracy: a discussion of Scandinavian research on system development. Scand J Inf Syst.

[31] Kensing F, Greenbaum J, Simonsen J, Robertson T (2012). Heritage: Having a say. Routledge international handbook of participatory design.

[32] Elovaara P, Igira FT, Mörtberg C, editors. Whose participation? whose knowledge?: exploring PD in Tanzania-Zanzibar and Sweden. Proceedings of the ninth conference on Participatory design: Expanding boundaries in design-Volume 1.; 2006: ACM.

[33] Brandt E, Binder T, Sanders EB-N, Simonsen J, Robertson T (2012). Tools and techniques: ways to engage telling, making and enacting. Routledge international handbook of participatory design.

[34] Rodwell CM (1996). An analysis of the concept of empowerment. J Adv Nurs.

[35] Feste C, Anderson RM (1995). Empowerment: from philosophy to practice. Patient Educ Couns.

[36] Funnell MM, Anderson RM (2004). Empowerment and self-management of diabetes. Clinical diabetes.

[37] Mol A. The logic of care: Health and the problem of patient choice. Routledge; 2008.

[38] Dubois C-A, Singh D, Jiwani I. The human resource challenge in chronic care. Caring for People with Chronic Conditions: A Health System Perspective, European Observatory on Health Systems and Policies Series, Berkshire. 2008:143–71.

[39] Melander-Wikman A, Jansson M, Ghaye T (2006). Reflections on an appreciative approach to empowering elderly people, in home healthcare. Reflective Practice: International and Multidisciplinary Perspectives.

[40] Jansson M, Mörtberg C. A Cup of Coffee: Users’ Needs and Experiences. Human-Centered Design of E-Health Technologies: Concepts, Methods and Applications. 2010:253.

[41] World Medical Association Declaration of Helsinki. Ethical principles for medical research involving human subjects [Online]. Available: http://www.wma.net/en/30publications/10policies/b3/: Accessed [2012–03-20]; 2013. 10.1001/jama.2013.281053 24141714

[42] Reason P, Bradbury H (2008). The SAGE handbook of action research : participative inquiry and practice.

[43] Lichtman M (2013). Qualitative research in education : a user’s guide.

[44] Coulson NS (2013). How do online patient support communities affect the experience of inflammatory bowel disease? An online survey. JRSM short reports.

[45] Coulson NS, Smedley R, Bostock S, Kyle SD, Gollancz R, Luik AI (2016). The pros and cons of getting engaged in an online social community embedded within digital cognitive behavioral therapy for insomnia: survey among users. J Med Internet Res.

[46] Malik S, Coulson NS (2010). ‘They all supported me but I felt like I suddenly didn’t belong anymore’: an exploration of perceived disadvantages to online support seeking. Journal of Psychosomatic Obstetrics & Gynecology.

